# S01-1 Using data and analytics to fully understand the barriers, and motivations to physical activity within a city setting

**DOI:** 10.1093/eurpub/ckac093.002

**Published:** 2022-08-29

**Authors:** Chris Scott

**Affiliations:** Communications, London Sport, London, United Kingdom

**Keywords:** Urbanisation, Data and insight, decision making, collaboration

## Abstract

**Issue/problem:**

By 2050, 68% of the world's population will live in cities. With increasing urbanisation there is a need for policymakers to fully understand the current situation within their society when it comes to being physically active.

**Description of the problem:**

In 2018, four global cities began a collaborative approach based on data and analytics to identify the barriers, and motivations to physical activity within a city setting. They needed to answer three questions:

- Who currently benefits the most from physical activity and why?
- What is the impact of existing policies and interventions?
- Where are the opportunities to maximize physical activity and its potential value?

The challenge is to understand to what extent inactivity is due to supply constraints that can be addressed by making physical activity more attractive and accessible, vs. demand constraints-perceived lack of time or being afraid to participate.

**Results (effects/changes):**

Using population survey information and databases on the locations of programmes, facilities and green space, our analysis has proven what works and what doesn't in each of the cities. For example, ethnic groups with lower levels of physical activity show higher levels of motivation to be active than other groups. Suggesting that lack of interest is not the reason for lower participation. We also found clear evidence of the impact and importance of facility access. Across all cities, access to facilities is correlated with levels of activity - with more active areas on average having almost 2.5 times more facilities than less active areas.

**Lessons:**

There are 3 learnings from this project:

- Data and insight -by using an evidence-based approach, policymakers have ensured that when resources and space are limited, they know how to create the highest value
- Collaboration - cities have led knowledge sharing through bi-lateral meetings, quarterly city calls and virtual working sessions to dissect and discuss comparative findings
- The value of physical activity - cities now understand the greater social impact of physical activity and are using this to build the case for further investment

**Main messages:**

A collaboration using data and analytics and best-practice sharing has enabled policymakers to apply evidence-led decision-making and have tangible impact on the lives of their citizens.

